# Downregulation of miR-30b-5p Facilitates Chondrocyte Hypertrophy and Apoptosis via Targeting Runx2 in Steroid-Induced Osteonecrosis of the Femoral Head

**DOI:** 10.3390/ijms231911275

**Published:** 2022-09-24

**Authors:** Lishan Lin, Yaling Yu, Kangping Liu, Yixin Jiang, Zhenlei Zhou

**Affiliations:** College of Veterinary Medicine, Nanjing Agricultural University, Nanjing 210095, China

**Keywords:** steroid-induced osteonecrosis of the femoral head, methylprednisolone, non-coding RNA, hypertrophic differentiation, apoptosis

## Abstract

As a widely used steroid hormone medicine, glucocorticoids have the potential to cause steroid-induced osteonecrosis of the femoral head (SONFH) due to mass or long-term use. The non-coding RNA hypothesis posits that they may contribute to the destruction and dysfunction of cartilages as a possible etiology of SONFH. MiR-30b-5p was identified as a regulatory factor in cartilage degeneration caused by methylprednisolone (MPS) exposure in our study through cell transfection. The luciferase reporter assay confirmed that miR-30b-5p was downregulated and runt-related transcription factor 2 (Runx2) was mediated by miR-30b-5p. The nobly increased expression of matrix metallopeptidase 13 (MMP13) and type X collagen (Col10a1) as Runx2 downstream genes contributed to the hypertrophic differentiation of chondrocytes, and the efficiently upregulated level of matrix metallopeptidase 9 (MMP9) may trigger chondrocyte apoptosis with MPS treatments. The cell transfection experiment revealed that miR-30b-5p inhibited chondrocyte hypertrophy and suppressed MPS-induced apoptosis. As a result, our findings showed that miR-30b-5p modulated Runx2, MMP9, MMP13, and Col10a1 expression, thereby mediating chondrocyte hypertrophic differentiation and apoptosis during the SONFH process. These findings revealed the mechanistic relationship between non-coding RNA and SONFH, providing a comprehensive understanding of SONFH and other bone diseases.

## 1. Introduction

Glucocorticoids (GC) are steroid hormones that are commonly utilized to treat various inflammatory diseases, autoimmune disorders, and cancer. However, inappropriate GC therapy is likely to have a number of negative effects on the endocrine, cardiovascular, musculoskeletal, and other systems [1]. Steroid-induced osteonecrosis of the femoral head (SONFH) is a metabolic disorder caused by improper GC medication that results in bone marrow destruction in the femoral head and hip joint impairment, which were initially documented in 1957 [2,3]. Presently, along with the widespread application of GC treatments, SONFH has garnered substantial attention in the medical field due to the challenges in regenerating osteonecrotic tissue. However, more evidence is required to investigate the precise pathogenesis of SONFH, which may be linked to several theories, such as lipid metabolism disorders, decreased osteogenesis capacity of bone marrow mesenchymal stem cells, inadequate blood supply, inflammation and cell apoptosis, and non-coding RNA and gene polymorphism [3].

Non-coding RNAs (ncRNAs) are a vital component in cell proliferation, growth, and death [4]. MicroRNAs (miRNAs) are short single-stranded ncRNAs that modulate target gene expression by inhibiting the translational regulation of target proteins. Several studies using microarray RT-PCR were conducted to investigate the potential relationship between miRNAs and SONFH, indicating that upregulated or downregulated miRNAs may act as a regulatory factor in the SONFH process [5,6]. MicroRNA-17/20a, which targets nuclear factor-kappa B ligand (RANKL) expression, was found to be downregulated in glucocorticoid-induced osteoclast differentiation [7]. In GC-inhibited osteoblast proliferation, microRNA-199a-5p functioned by regulating the WNT signaling pathway [8]. This evidence aided the discovery of the mechanism of SONFH.

In 1997, runt-related transcription factor 2 (Runx2) was initially identified as a critical transcription factor in skeletal development [9,10]. Numerous studies have discovered that Runx2 plays an important role in multipotent mesenchymal cells, osteoblast-lineage cells, and chondrocytes with a variety of functions [11]. Runx2 was expressed weakly in uncommitted mesenchymal cells in osteoblasts, but it was elevated in preosteoblasts, had its peak expression in immature osteoblasts, and was eventually downregulated in mature osteoblasts [12]. Runx2 regulates the bone matrix genes osteocalcin (OCN), bone sialoprotein (BSP), and osteopontin (OPN), and it maintains the expression of OPN and BSP during the osteoblast differentiation process [13]. In chondrocytes, Runx2 is upregulated in prehypertrophic chondrocytes and remains upregulated in hypertrophic and terminal hypertrophic chondrocytes [14]. Matrix metallopeptidase 9 (MMP9) and 13 (MMP13) are zinc-containing endopeptidases that can degrade a variety of extracellular matrix proteins [15]. Type X collagen (Col10a1) is a member of the short-chain collagen family that participates in calcification via matrical organization changes [16]. MMP13 and Col10a1 have been identified as downstream targets of Runx2 as hypertrophic chondrocyte markers [17,18].

Several functional studies have been carried out to investigate the biological role of miR-30b-5p in acute pancreatitis, renal cell carcinoma, and lung cancer [19,20,21]. However. few studies have been conducted to explore the interaction between miR-30b-5p and Runx2. Nurzati et al. found that the abnormal expression of Ruxn2 in keloids may be regulated by miR-30b-5p in the pathogenesis of keloids based on the GEO database [22]. Moreover, miR-30b-5p has been proved to be a tumor suppressor microRNA in esophageal squamous cell carcinoma by targeting Runx2 [23].

The current research revealed the role of miR-30b-5p in chondrocytes during SONFH targeting Runx2. Transfection experiments revealed that miR-30b-5p was reduced in a femoral head necrosis model, and the target gene Runx2 was confirmed. The elevated Runx2 level was able to stimulate the expression of MMP13 and Col10a1 related to chondrocyte hypertrophy alterations, as well as chondrocyte apoptosis possibly mediated by MMP9. Our findings are beneficial for investigating the function and mechanism of ncRNAs in SONFH and other bone diseases.

## 2. Results

### 2.1. Methylprednisolone (MPS) Exposure Led to Chondrocyte Hypertrophy and Apoptosis in the SONFH Animal Model

**Figure 1:**
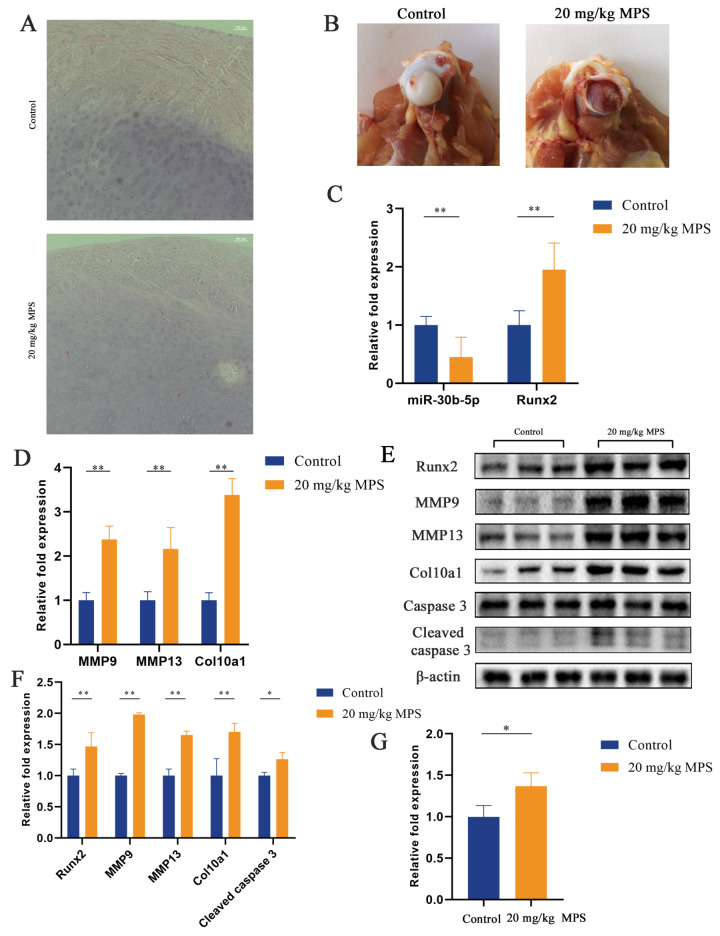
Chondrocyte hypertrophy and apoptosis were trigged by MPS exposure in SONFH animal model. (**A**) Hematoxylin and eosin (H&E) staining of chondrocytes in the femoral head articular cartilage. Scale bar: 200 µm. Group 1: animals injected with sterile saline; Group 2: animals injected with methylprednisolone (MPS) (20 mg/kg/d); (**B**) The macroscopic morphological changes in control group and MPS-induced group. (**C**,**D**) The expression levels of miR-30b-5p, Runx2, MMP9, MMP13, and Col10a1 were determined by qPCR between the control group and MPS group. (**E**,**F**) The protein expression of Runx2, MMP9, MMP13, and Col10a1 detected by western blotting analysis between the control group and MPS group. (**G**) The apoptosis of chondrocytes was performed by caspase 3 activity kit. Data were presented as mean ± SD. “*” represented statistical difference in comparison with control group (* *p* < 0.05, ** *p* < 0.01).

Chondrocyte hypertrophy and apoptosis in the SONFH animal model have been evaluated by hematoxylin and eosin (H&E) staining, quantitative real-time PCR (qPCR), western blotting, and a caspase 3 activity kit. The result of H&E staining is shown in Figure 1A, focusing on the femoral head articular cartilage. Chondrocytes in the control group exhibited normal morphology, while abnormal hypertrophic chondrocytes were observed in the MPS-induced group (red arrows). The separation of the cartilage from the femoral head was seen in the MPS group, indicating the destruction and dysfunction of the cartilage (Figure 1B). The qPCR test revealed a significant decrease in miR-30b-5p with a markedly elevated level of hypertrophic-related genes Runx2, MMP9, MMP13, and Col10a1 (Figure 1C,D). The results of the qPCR assay were confirmed by the western blotting data (Figure 1E,F). Furthermore, chondrocyte apoptosis was assessed using a western blotting analysis and a caspase 3 activity kit. The results demonstrated that cleaved caspase 3 was upregulated by MPS exposure, indicating the apoptosis of chondrocytes by the MPS treatment (Figure 1E–G). Overall, the results indicated that MPS exposure induced hypertrophic differentiation and apoptosis in chondrocytes in the SONFH animal model.

### 2.2. Effects of MPS Exposure on Hypertrophic Differentiation and Apoptosis in Chondrocytes

**Figure 2:**
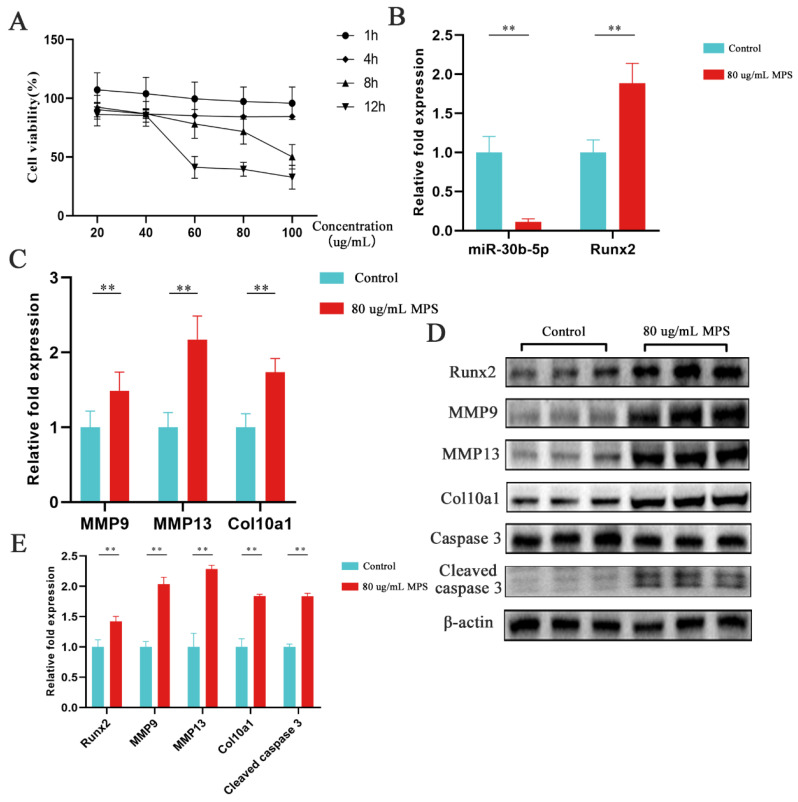
MPS exposure promoted hypertrophy differentiation in chondrocytes. (**A**) The cell viability of chondrocytes treated with 20, 40, 60, 80, 100 µg/mL for 1, 4, 8, 12 h. (**B**,**C**) The expression of miR-30b-5p, Runx2, MMP9, MMP13, and Col10a1 was determined by quantitative real-time PCR. Group 1: control; Group 2: cells treated with 80 µg/mL methylprednisolone (MPS). (**D**,**E**) the protein expression of Runx2, MMP9, MMP13, Col10a1, and Cleaved caspase 3 in MPS-induced chondrocytes compared with control group. Data were presented as mean ± SD. “*” represented statistical difference in comparison with control group (** *p* < 0.01).

**Figure 3:**
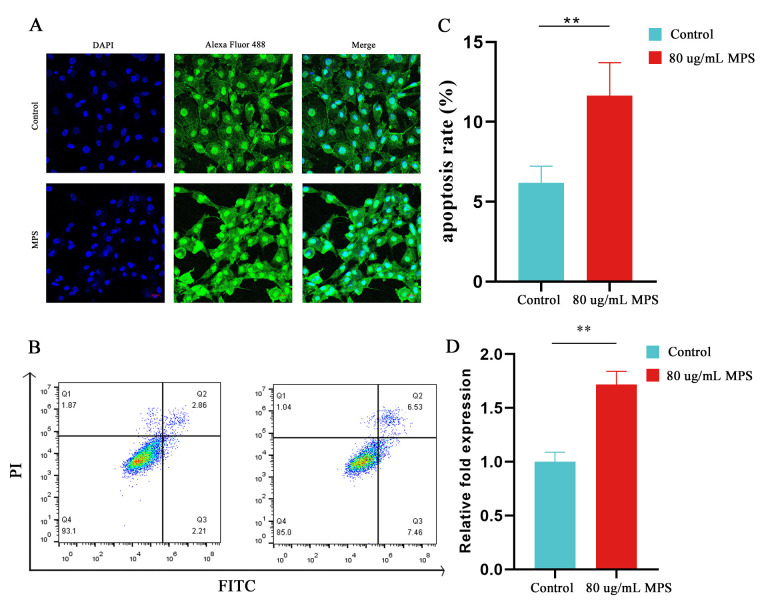
MPS-induced chondrocytes apoptosis in vitro. (**A**) Runx2 expression stained with Alexa Fluor 488 fluorescence (green) was visualized by immunofluorescence, and nuclei were stained with DAPI (blue). Group 1: control; Group 2: cells treated with 80 µg/mL methylprednisolone (MPS). Scale bar: 20 µm. (**B**,**C**) Cells were stained with PI and Annexin V-FITC and detected by flow cytometry. (**D**) The caspase 3 activity between control group and MPS group. Data were presented as mean ± SD. “*” represented statistical difference in comparison with control group (** *p* < 0.01).

The hypertrophic differentiation and apoptosis in chondrocytes have been evaluated by qPCR, western blotting, immunofluorescence, flow cytometry, and a caspase 3 activity kit to strengthen the observation in chondrocytes with MPS treatment in the animal models. Chondrocytes were exposed to various doses of MPS (20, 40, 60, 80, and 100 µg/mL) for 1, 4, 8, and 12 h, respectively, to evaluate the cytotoxicity of MPS. As displayed in Figure 2A, the cell viability of chondrocytes was reduced by around 30% under 80 ug/mL MPS exposure for 8 h. As a result, the condition was chosen for further in vitro study. Firstly, miR-30b-5p was determined using qPCR, showing that it was dramatically downregulated with the MPS treatment, whereas the mRNA levels of Runx2, MMP9, MMP13, Col10a1, and cleaved caspase 3 were increased inversely (Figure 2B,C). Moreover, the protein levels of Runx2, MMP9, MMP13, Col10a1, and cleaved caspase 3 were enhanced (Figure 2D,E). The immunofluorescence assay result was consistent with the western blotting assay, indicating that Runx2 expression was increased in MPS-induced chondrocytes compared to the control group (Figure 3A). A caspase 3 activity kit and a flow cytometry experiment were used. The results showed that the MPS group exhibited significantly higher apoptosis rates and enhanced caspase 3 activity, as shown in Figure 3B–D. In conclusion, MPS exposure significantly increased the expression of Runx2 and hypertrophic marker genes MMP13 and Col10a1.

### 2.3. MiR-30b-5p Regulated Chondrocyte Hypertrophy with MPS Treatment in Chondrocytes

**Figure 4:**
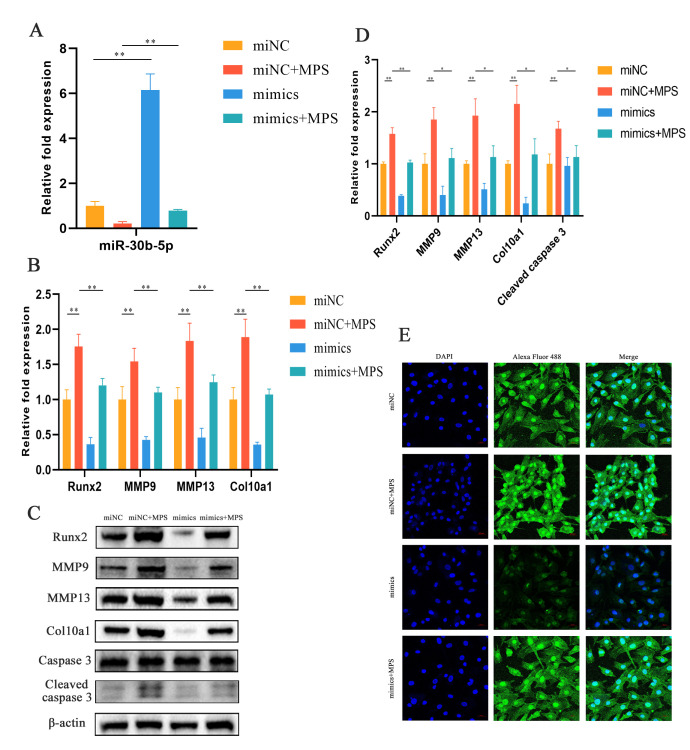
The overexpression of miR-30b-5p inhibited the hypertrophic differentiation in chondrocytes. (**A**,**B**) The expression of miR-30b-5p, Runx2, MMP9, MMP13, and Col10a1 was performed by qPCR with miR-30b-5p transfection. Group 1: mimics negative control (miNC); Group 2: miNC and methylprednisolone (MPS); Group 3: mimics; Group 4: mimics and MPS. (**C**,**D**) The protein expression of Runx2, MMP9, MMP13, and Col10a1 detected by western blotting assay in the four groups mentioned above. (**E**) Runx2 was visualized by Alexa Fluor 488 (green) and the nuclei was visualized by DAPI (blue) through immunofluorescence analysis. Scale bar: 20 µm. Data were presented as mean ± SD. “*” represented statistical difference in comparison with control group (* *p* < 0.05, ** *p* < 0.01).

The association between MPS and miR-30b-5p in chondrocytes has been evaluated by qPCR, western blotting, and immunofluorescence with cell transfection. The transfection of miR-30b-5p mimics or mimics negative control (NC) was performed with or without MPS exposure. As demonstrated in Figure 4A,B, the transfection of miR-30b-5p mimics effectively reduced Runx2 expression with a nobly increased level of miR-30b-5p. Furthermore, the elevated Runx2 was significantly suppressed compared to mimics NC with the MPS treatment. In terms of qPCR and western blotting assays, the expression levels of MMP9, MMP13, and Col10a1 as Runx2 downstream genes were consistent (Figure 4B–D). The immunofluorescence results confirmed the opposite effect of miR-30b-5p mimics’ transfection by MPS exposure on the Runx2 levels (Figure 4E). In conclusion, these findings indicated that miR-30b-5p regulated Runx2 expression, which is linked to hypertrophic differentiation.

### 2.4. MPS-Induced Apoptosis in Chondrocytes Was Reduced by miR-30b-5p

**Figure 5:**
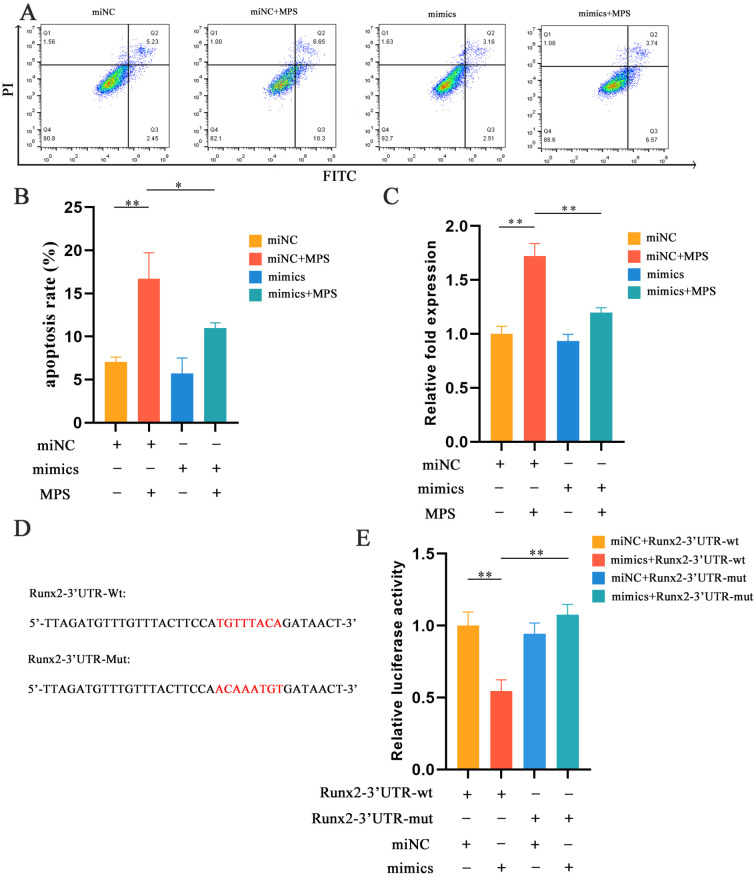
miR-30b-5p suppressed MPS-induced apoptosis in chondrocyte. (**A**,**B**) The apoptosis of chondrocytes with different treatments were stained with Annexin V–FITC and PI by flow cytometry assay. Group 1: mimics negative control (miNC); Group 2: miNC and methylprednisolone (MPS); Group 3: mimics; Group 4: mimics and MPS. (**C**) The bar chart showed the caspase 3 activities of different treatment groups by a caspase 3 activity kit. (**D**) The sequence of Runx-3′UTR-wt and Runx2-3′UTR-mut was determined by TargetScan Release 7.1. (**E**) The luciferase activity in the group with Runx2-3′UTR-wt and miR-30b-5p mimics was significantly decreased compared with the group that combined Runx2-3′UTR-wt and miNC. Data were presented as mean ± SD. “*” represented statistical difference in comparison with control group (* *p* < 0.05, ** *p* < 0.01).

The role of miR-30b-5p in apoptosis has been evaluated by western blotting, flow cytometry, and a caspase 3 activity kit with cell transfection. MiR-30b-5p mimics or mimics NC were transfected into chondrocytes with or without the MPS treatment and the western blotting analysis revealed that miR-30b-5p mimics alone did not affect apoptosis, but miR-30b-5p mimics’ transfection significantly suppressed apoptosis caused by MPS exposure. Furthermore, a flow cytometry analysis and a caspase 3 activity kit were used to further investigate the relationship between miR-30b-5p and apoptosis. The above experiments revealed the same phenomenon (Figure 5A–C). Overall, these findings suggested that miR-30b-5p had the capacity to alleviate apoptosis induced by MPS.

**Figure 6:**
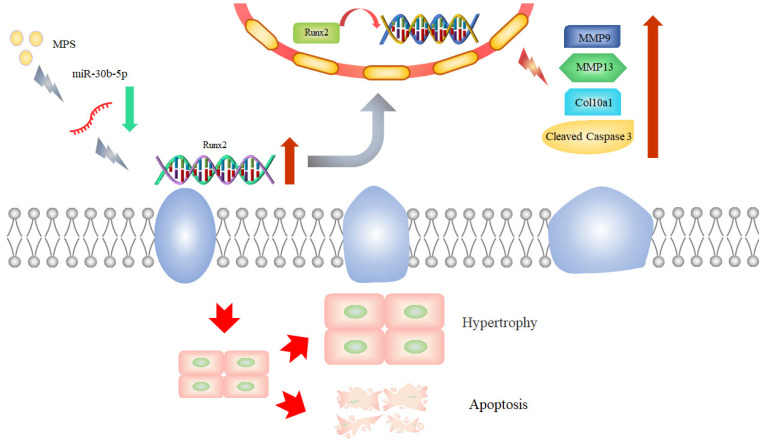
The schematic diagram of the mechanism.

The link between miR-30b-5p and Runx2 has been evaluated by a luciferase reporter experiment. The results showed that miR-30b-5p significantly reduced the luciferase activity of the wild-type plasmid, while there was no significant change in the mutant-type group (Figure 5D,E). Runx2 was found to be regulated by miR-30b-5p and to mediate chondrocyte hypertrophy and apoptosis. Figure 6 depicts a schematic diagram of the mechanism.

## 3. Discussion

As a bone metabolic abnormality, SONFH is characterized by peri-articular bone disintegration and cartilage deterioration [24]. SONFH’s high impairment rate with a poor quality of life is a major concern [25]. Scholars worldwide have been striving to establish animal models of SONFH pathogenesis, including rabbits, mice, rats, and chickens [26,27,28]. The advantage of chickens as an animal model of SONFH over other small quadrupeds is that their bipedal movement produces pressures on their hips similarly to humans [29]. The first SONFH chicken model was established using weekly intramuscular injections of 3 mg/kg MPS with an ONFH result of the resorption of subchondral bone death, fat cell proliferation, and new bone growth [30]. Erken et al. discovered that chickens were suitable for steroid-induced osteonecrosis models because similar clinical changes were observed by injecting MPS (3 mg/kg/week) intramuscularly [31].

The downregulation of miR-30b-5p in our study was confirmed by the significantly increased expression of Runx2, MMP9, MMP13, and Col10a1 in the SONFH animal model. Several functional studies on miR-30b-5p in vertebrates have been conducted. It was discovered that miR-30b-5p-targeted LRP8 re-sensitizes lung cancer cells to DDP, acting as a tumor suppressor [19]. Moreover, the research suggested that miR-30b-5p modulated autophagy and lysosomal biogenesis by blocking TFEB-dependent transactivation via CLEAR elements in the nucleus [32]. Furthermore, it was demonstrated that exosomal miR-30b-5p produced by hypoxic PDAC cells increased angiogenesis by inhibiting GJA1 and that miR-30b-5p could be used to diagnose PDAC [33]. The role of miR-30b-5p regulation in SONFH, however, remains unknown. In our study, miR-30b-5p was significantly reduced after MPS exposure both in vivo and in vitro, and its target gene Runx2 was identified using a dual luciferase reporter assay. Runx2 was found to be a regulatory factor in the expression of MMP13 and Col10a1 in hypertrophic chondrocytes [34]. Although the expression of MMP13 is beneficial to normal cartilage remodeling, excessive MMP13 activity may lead to an aberrant matrix breakdown and joint degeneration [35]. As a physiological process, hypertrophic differentiation occurs exclusively in growth plate chondrocytes during endochondral ossification [36]. However, the significantly elevated expression levels of Runx2, MMP13, and Col10a1 in the femoral head articular cartilage suggested that the abnormal hypertrophic differentiation occurred in chondrocytes and may result in cartilage destruction and dysfunction during the SONFH process.

Apoptosis is a common type of programmed cell death used to maintain an internal environment. This study identified in vitro and in vivo chondrocyte apoptosis in response to MPS exposure. A prior study showed that MMP9/gelatinase B deficient homozygous mice exhibited normal hypertrophic chondrocytes with delayed apoptosis vascularization and ossification, indicating that gelatinase B may function to induce chondrocyte apoptosis [37]. MMP9 expression was inhibited in Runx2-deficient chondrocytes, indicating that MMP9 is the downstream regulator of Runx2, according to Liao et al. [38]. In addition, Rashid et al. [39] found that the apoptosis of Runx2HC/HC hypertrophic chondrocytes was significantly reduced, as was the level of MMP9. The extraordinarily elevated expression of MMP9 controlled by Runx2 may contribute to the induction of apoptosis during the SONFH process.

In conclusion, we discovered that miR-30b-5p was significantly decreased with MPS exposure in vitro and in vivo, accompanied by Runx2 being significantly upregulated. As a transcription factor, the significantly increased expression of Runx2 facilitated hypertrophic differentiation by activating the expression of MMP13 and Col10a1 and induced chondrocyte apoptosis by possibly stimulating the level of MMP9 during the development of SONFH. Our findings provided a new investigation into the role of ncRNAs as a biological regulator in the mechanism of SONFH.

## 4. Materials and Methods

### 4.1. Femoral Head Necrosis Model Animals

On average, 16 one-day-old broiler chickens (*Gallus gallus*, AA broilers) were randomly assigned to two groups: control and MPS. At 29 days, the animals were given MPS (20 mg/kg/d) intramuscularly for a week, while the control group received sterile saline injections. Animal samples from each group were collected at 42 days for further research. All animal protocols were approved by the Animal Protection and Use Committee of Nanjing Agricultural University (approval number: No.2019031804).

### 4.2. Cell Culture, Drug Stimulation, Cell Viability Assay

The isolation of chondrocytes was carried out as previously described [26]. Chondrocytes were treated with MPS at different doses (20, 40, 60, 80, 100 µg/mL) and times (1, 4, 8, 12 h) to determine cell viability using a Cell Counting Kit-8 (CCK-8) (Cat No. BS350A, Biosharp, Hefei, China). A Tecan Spark Multimode Microplate Reader (Tecan, Männedorf, Switzerland) was used to measure absorbance values at 450 nm, according to the manufacturer’s instructions.

### 4.3. RNA Isolation and Quantitative Real-Time PCR

Total RNA was isolated from tissues and cells using an RNA isolater (Cat No. R401-01, Vazyme, Nanjing, China), according to the manufacturer’s instructions. HiScript III All-in-one RT SuperMix Perfect for qPCR (Cat No. R333-01, Vazyme, China) was used to make cDNA, and Taq Pro Universal SYBR qPCR Master Mix (Cat No. Q712, Vazyme, China) was used to perform qPCR on an ABI PRISM 7300 HT sequence-detection system (Applied Biosystems Inc., Waltham, MA, USA).

### 4.4. Western Blotting

Western blotting analysis was conducted as previously reported [40]. Anti-Runx2 (Cat No. WL03358, 1:500, WanleiBio, Shenyang, China), anti-MMP9 (Cat No. WL03096, 1:500, WanleiBio, China), anti-MMP13 (Cat No. WL04694, 1:500, WanleiBio, China), anti-Col10a1 (Cat No. AF6538, 1:500, Beyotime, Shanghai, China), anti-Caspase 3 (Cat No. WL02117, 1:500, WanleiBio, China), anti-Cleaved caspase 3 (Cat No. WL01992, 1:500, WanleiBio, China), anti-β-actin (Cat No. 20536-1-AP, 1:1000, Proteintech, Wuhan, China) primary antibodies were used.

### 4.5. Immunofluorescent Staining

Following various treatments, the cells cultivated on coverslips were stained using immunofluorescence. In brief, the cells were fixed for 20 min in 4% paraformaldehyde before being washed three times in PBS for 3 min. The cells were then permeabilized for 20 min with 0.3% Triton X-100 and rinsed three times with PBS for 3 min. The cells were blocked with 5% BSA for 30 min before being treated at 4 °C for 14 h with anti-rabbit polyclonal antibodies to Runx2 (Cat No. 20700-1-AP, 1:100, Proteintech, China). The cells were then rinsed three times with PBS containing 0.1% tween-20 (PBST) for 3 min before being incubated for 1 h with an Alexa Flour 488 goat anti-rabbit antibody (Cat No. P0176, 1:1000, Beytime, China). Finally, the cells were rinsed with PBST three times for 3 min and incubated with 4,6-diamidino-2-phenylindole (DAPI) for 5 min before being washed with PBST four times for 5 min. An Antifade Mounting Medium was used to secure the coverslips to the glass slides (Cat No. FSL004, Beyotime, China). An LSM 710 confocal laser microscope equipment was used to view the images (Zeiss, Jena, Germany).

### 4.6. Flow Cytometry

The Annexin V-FITC/PI Apoptosis test was used to detect apoptosis (Cat No. A211-01, Vazyme, China). In brief, each group’s chondrocytes were rinsed with PBS and digested with 0.25% trypsin without EDTA. The cells were then resuspended in PBS before being centrifuged twice at 1000 rpm per minute for 5 min. Finally, Annexin V-FITC and PI staining solutions were added and incubated for 10 min in the dark before being measured on a BD FACSVerseTM 273 Flow Cytometer (BD Biosciences, Franklin Lakes, NJ, USA)

### 4.7. Measurement of Caspase-3 Activity

A caspase 3 activity kit (Cat No. C1115, Beyotime, China) was used to detect caspase 3 activity. Caspase 3 has the ability to catalyze the substrate acetyl-Asp-Glu-Val-Asp p-nitroaniline (Ac-DEVD-pNA) to create yellow p-nitroaniline (pNA), as measured by a Tecan Spark Multimode Microplate Reader at 405 nm (Tecan, Switzerland).

### 4.8. Cell Transfection

The miR-30b-5p mimics were chemically synthesized for overexpression (GenePharma, Shanghai, China). The experimental groups included 4 groups: (1) negative control transfection; (2) MPS exposure after negative control transfection; (3) miR-30b-5p mimics overexpression; and (4) miR-30b-5p mimics overexpression followed by MPS exposure. Cells were transfected with RFect Transfection Reagent (Cat No. 11014, BIOG, Changzhou, China) as per the manufacturer’s instructions. Cells were used for the further experiment after transfection for 48 h.

### 4.9. Dual Fluorescence Assay

To confirm the link between Runx2 and miR-30b-5p, a dual fluorescence test was performed. The wild-type and mutant 3’UTRs of Runx2 mRNA were cloned and inserted into the GP-miRGLO vector (Promega, Madison, WI, USA) and co-transfected with miR-30b-5p mimics or miR-NC into chondrocytes. Tecan Infinite M200 Pro NanoQuant (Tecan, Switzerland) was utilized to assess firefly and Renilla luciferase activity after transfection for 48 h using the Dual Luciferase Reporter Assay Kit (Cat No. DL101-01, Vazyme, China).

### 4.10. Statistical Analysis

The IBM SPSS 25.0 program for Windows was used to analyze all of the results (IBM Inc., Armonk, NY, USA). Data for each group were represented as mean SD values, and differences between groups were assessed using one-way ANOVA (ANOVA, LSD). Significant changes were assessed at two levels of significance: *p* < 0.05 (significant) and *p* < 0.01 (extremely significant).

## Data Availability

No new data were created or analyzed in this study. Data sharing is not applicable to this article.
